# Wireless Optogenetic Microsystems Accelerate Artificial Intelligence–Neuroscience Coevolution Through Embedded Closed-Loop System

**DOI:** 10.3390/mi16050557

**Published:** 2025-05-03

**Authors:** Sungcheol Hong

**Affiliations:** Department of Electronic & Electrical Convergence Engineering, Hongik University, Sejong 30016, Republic of Korea; hyhaerong@hongik.ac.kr

**Keywords:** neuroscience, artificial intelligence, wireless optogenetics, neurotechnology, brain, closed-loop neural interface

## Abstract

Brain-inspired models in artificial intelligence (AI) originated from foundational insights in neuroscience. In recent years, this relationship has been moving toward a mutually reinforcing feedback loop. Currently, AI is significantly contributing to advancing our understanding of neuroscience. In particular, when combined with wireless optogenetics, AI enables experiments without physical constraints. Furthermore, AI-driven real-time analysis facilitates closed-loop control, allowing experimental setups across a diverse range of scenarios. And a deeper understanding of these neural networks may, in turn, contribute to future advances in AI. This work demonstrates the synergy between AI and miniaturized neural technology, particularly through wireless optogenetic systems designed for closed-loop neural control. We highlight how AI is now revolutionizing neuroscience experiments from decoding complex neural signals and quantifying behavior, to enabling closed-loop interventions and high-throughput phenotyping in freely moving subjects. Notably, AI-integrated wireless implants can monitor and modulate biological processes with unprecedented precision. We then recount how neuroscience insights derived from AI-integrated neuroscience experiments can potentially inspire the next generation of machine intelligence. Insights gained from these technologies loop back to inspire more efficient and robust AI systems. We discuss future directions in this positive feedback loop between AI and neuroscience, arguing that the coevolution of the two fields, grounded in technologies like wireless optogenetics and guided by reciprocal insight, will accelerate progress in both, while raising new challenges and opportunities for interdisciplinary collaboration.

## 1. Introduction

Modern AI owes many of its foundational ideas to neuroscience. The very notion of an artificial “neural network” arose from abstracting how biological neurons connect and learn. Early pioneers like McCulloch, Pitts, and Rosenblatt were directly inspired by brain anatomy and function [1,2,3]. For instance, the concept of Hebbian learning, “cells that fire together wire together” from neuroscience, underpinned the first artificial neural networks [3,4]. This simple network of weighted connections was an attempt to mimic how a brain might learn to recognize patterns [5,6]. Over subsequent decades, neuroscientific principles continued to guide AI. The brain’s layered visual processing hierarchy inspired the design of convolutional neural networks (CNNs) for image recognition, and its working memory capacity motivated recurrent neural networks (RNNs) that feed outputs back as inputs to handle sequences [7,8,9]. Even the trial-and-error learning animals use to seek rewards laid the groundwork for reinforcement learning algorithms in AI. In short, many milestones in AI, from deep learning architectures to learning rules, trace their lineage to insights about how real neural circuits compute and adapt.

Yet, the influence was not one-way. As AI and computer science advanced, they provided formal models and tools that helped neuroscientists frame hypotheses about the brain [10,11,12,13]. The earliest artificial networks were simplistic compared to biological brains, but they demonstrated that brain-inspired computation could yield intelligent behavior [14]. This symbiosis has only deepened with time: neuroscientists draw on AI models to interpret cognition, while AI researchers turn to neural processing for inspiration on making machines more brain-like [15]. The stage was set for a reciprocal relationship, one that has evolved into a virtuous cycle of innovation in recent years.

## 2. AI Empowering Neuroscience, Wireless Optogenetics, and Beyond

**Figure 1:**
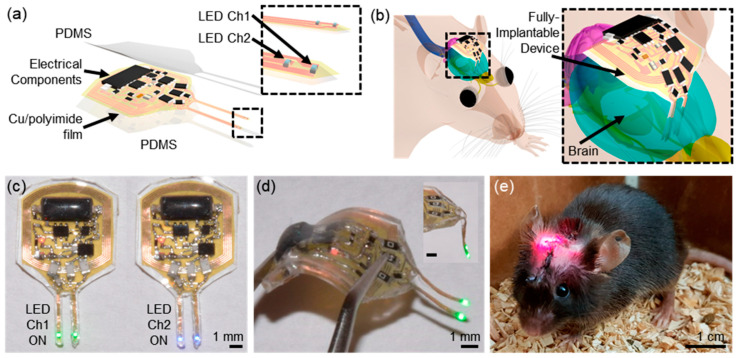
System overview. (**a**) Schematic illustration of a soft, fully implantable dual-channel optogenetic device. (**b**) Illustration of the device location relative to the brain. (**c**) Demonstration of a dual-channel wireless operation. (**d**) Picture of the device after bending the body of the device. (**e**) Image of a mouse with the device implanted [20].

Today, AI is propelling neuroscience into a new era of data-driven discovery and precise intervention. In particular, the convergence of AI with wireless optogenetics, untethered from cables, is enabling experiments once unimaginable [16,17,18,19,20,21,22,23,24]. By coupling intelligent algorithms with miniaturized, implantable devices, researchers can decode neural activity [25], analyze behavior [26,27], and even trigger stimuli in real time [28], all in freely moving animals. This suggests the possibility of truly closed-loop neuroscience experiments, where experimental conditions could autonomously adjust based on feedback [29]. Wireless optogenetic platforms represent a class of miniaturized, implantable neurotechnologies that enable high-precision, untethered stimulation and the recording of neural circuits (Figure 1) [20].

**Figure 2:**
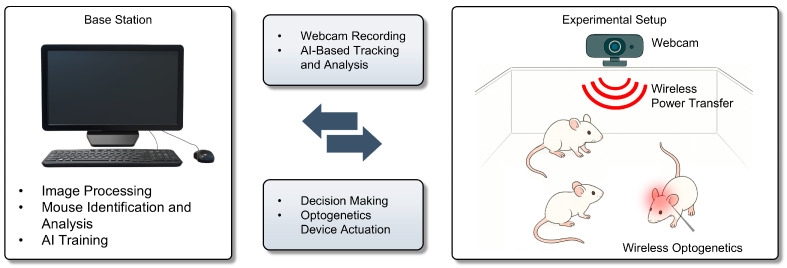
Closed-loop wireless optogenetic system driven by real-time behavior tracking and AI-based decision making.

One groundbreaking example is the development of AI-assisted wireless implants for photodynamic therapy and neuromodulation. An implantable, multichannel optoelectronic device was recently created that can be implanted with ease and used to deliver controlled light for tumor treatment. Uniquely, this system integrates a deep neural network (the pose-tracking algorithm DeepLabCut) and a Monte Carlo light simulation [30,31]. In this experimental context, the primary role of AI is to enable the real-time identification and tracking of freely moving mice. While wireless optogenetic devices have successfully removed physical constraints on animal behavior, they have historically faced a key technical limitation in multi-animal experiments: the inability to accurately track and monitor multiple subjects simultaneously. While wireless optogenetic devices have successfully removed physical constraints on animal behavior, they have still faced a key technical limitation in multi-animal experiments, i.e., the challenge of accurately tracking and monitoring multiple subjects simultaneously. The integration of AI-based tracking algorithms, particularly deep learning-driven pose estimation, has now addressed this challenge, for the first time enabling scalable, closed-loop optogenetic experiments in freely behaving animals (Figure 2). These results underscore how embedded AI models, optimized for low-power wireless implants, enable scalable and autonomous neural interfacing under real-world constraints. Thus, researchers simultaneously controlled implants in multiple mice, demonstrating that AI can coordinate complex experiments at scale [32,33]. While developed for oncology, the underlying approach—AI-guided, wireless optical modulation—is directly transferrable to neuroscience experiments (for example, controlling neural circuits with multiple colors of light in many animals at once). It exemplifies how AI empowers precise, closed-loop optogenetic interventions. The system monitors conditions and immediately adjusts optical stimulation for optimal results. In parallel, AI is supercharging our ability to decode neural data and behavior [25,34]. Advances in machine learning enable us to sift through the massive streams of neural signals and video recordings that modern experiments generate [35,36]. For instance, deep learning models can decode a mouse’s brain activity to predict its next action, or conversely, analyze a video of an animal’s behavior to infer its internal neural states [25,37,38,39]. Tools like DeepLabCut (used in the example above) allow for the automated tracking of an animal’s posture and movements with human-level accuracy, providing quantitative readouts of behavior for correlation with neural recordings [30,31]. Such AI-driven behavior analysis is crucial for high-throughput phenotyping, assessing how neural circuit manipulations alter behavior across many individuals. Instead of a human observer scoring videos, a neural network can evaluate dozens of behaviors simultaneously and without bias. Moreover, wireless optogenetic implants typically operate below 10 mW during active stimulation phases, resulting in negligible tissue heating (a < 1 °C increase, confirmed via in vivo thermal imaging). Compared to traditional tethered optical fibers using external lasers (>100 mW at source), wireless systems dramatically reduce systemic power load while maintaining sufficient light delivery for effective neuromodulation.

Wireless optogenetic platforms augmented by AI are unlocking especially rich opportunities. Researchers have engineered soft, fully implantable optogenetic devices that deliver light inside specific organs or brain regions without tethering the animal. These platforms represent a class of miniaturized, intelligent neural interfaces that eliminate the need for cables while enabling precise, high-resolution control of brain activity. When combined with AI, these devices become smart systems. They can record physiological signals, run on-board algorithms to detect certain patterns, and trigger light stimulation accordingly (all powered and controlled wirelessly). Even in such situations, machine learning can be effectively utilized not only for tracking multiple subjects in a single cage but also for controlling animals housed individually across multiple cages. Furthermore, the study demonstrated that optogenetics can stimulate not only the brain but also the gut, highlighting that removing tethers enables the targeting of peripheral as well as central nervous systems. This suggests broader potential for expanding our understanding of neural networks beyond the brain [40,41]. Closed-loop control represents a powerful new frontier for AI in neuroscience. In these systems, neural activity or behavior is monitored in real time, and stimulation is dynamically adjusted based on AI-driven pattern recognition [29]. This approach has been shown to prevent seizures in rodent models by triggering targeted optical stimulation upon the detection of specific neural signatures. More broadly, AI enables the adaptive, real-time tuning of stimulation to maintain desired brain states, revealing circuit dynamics with far greater precision than manual methods. As a result, experiments increasingly feature AI not only as an observer but as an active modulator of neural function [42,43,44].

In sum, AI has moved from passive analysis to active experimentation. By decoding behavior and guiding interventions through wireless optogenetic implants, AI enables high-throughput, closed-loop studies that deepen our understanding of brain and peripheral systems. However, there are certainly technical details that need to be addressed. In current closed-loop systems, real-time video is processed externally via deep learning algorithms such as DeepLabCut. Behavioral classifications are transmitted wirelessly to control implants with a typical end-to-end latency of approximately 100–300 ms, primarily limited by video frame acquisition and RF communication delays. On-device computation remains challenging due to power constraints (<10 mW), so real-time inference is typically externalized, while implant devices respond autonomously based on decoded triggers.

## 3. Discoveries Looping Back, Neuroscience Inspiring Better AI

While artificial intelligence has advanced rapidly in recent years, several persistent limitations remain. Machine learning models typically rely on large-scale, labeled datasets and struggle with generalization under data-sparse conditions [45,46]. They often suffer from catastrophic forgetting when exposed to sequential learning tasks [47,48,49], and in generative systems such as large language models, they frequently produce factually incorrect but syntactically plausible outputs—a phenomenon known as hallucination [50]. Furthermore, these models are computationally intensive, requiring significant energy and hardware resources [51,52,53], in stark contrast to the brain’s compact, low-power operation. These shortcomings, however, present an opportunity; rather than treating neuroscience as merely inspirational, we can begin to use it as a source of empirical solutions. Advances in wireless optogenetics and AI-enabled tracking now allow for the real-time, closed-loop interrogation of neural circuits in freely behaving animals. These systems offer a platform for reverse-engineering biological intelligence under experimentally controlled conditions.

In other words, unresolved issues in current AI models, such as catastrophic forgetting, may potentially be addressed through a deeper understanding of neural circuits. Unlike today’s AI models, which rely on relatively simple neural connections, our actual brain circuits are composed of complex loops and mechanisms, including GABAergic inhibitory circuits [54,55]. Researchers can examine how the brain preserves previously learned information while encoding new inputs. Such experiments may uncover regulatory mechanisms, such as circuit-level gating or synaptic stabilization, that could be translated into architectural solutions for continual learning in artificial systems. Similarly, to address the issue of hallucination in AI, the optogenetic stimulation of prediction error circuits (e.g., the anterior cingulate cortex or orbitofrontal cortex) during ambiguous sensory decision-making tasks can reveal how the brain evaluates internal certainty and suppresses false inferences [56,57]. Incorporating such biological error-correction mechanisms could inform the design of AI systems capable of assessing their own output reliability, potentially leading to more trustworthy generative models. The data inefficiency problem can be explored through one-shot or few-shot learning paradigms in rodents, where wireless optogenetic systems are used to monitor and manipulate neuromodulatory inputs (such as dopamine or acetylcholine) during rapid associative learning. Observing how neuromodulators shape synaptic plasticity under minimal exposure may point toward biologically informed meta-learning strategies in AI. Finally, the brain’s energy-efficient computation, achieved through event-driven, sparse, and local processing, can be dissected through spatiotemporally patterned optogenetic activation of distributed networks. These experiments may identify core principles behind low-power neural coding, informing neuromorphic hardware design or the development of sparse, asynchronous AI architectures.

In sum, AI-powered neuroscience experiments are increasingly capable of uncovering not just how the brain functions but also how it solves the problems that modern AI still struggles with. This convergence enables us to shift from biologically inspired design to biologically validated design—where mechanisms discovered in vivo directly shape future machine learning algorithms. In doing so, we may begin to close the loop between brain and machine in the most literal sense.

## 4. Discussion and Future Outlook

The notion of biologically validated AI remains speculative and exploratory. Rather than aiming for exact translation, future research may focus on identifying abstract principles, such as hierarchical gating or local learning, from neuroscience that show functional benefits when implemented in AI. Discrepancies between neural and model behavior should not be seen as failures but as feedback points for refining both biological theory and computational modeling.

Nonetheless, the coevolution of neuroscience and artificial intelligence is no longer a theoretical idea—it is a demonstrable and accelerating process. From neural networks inspired by early models of the brain to today’s closed-loop wireless optogenetic systems empowered by AI, the relationship has matured into a bidirectional engine of innovation. This perspective has illustrated how AI now acts as both a lens and a lever: it not only helps us observe the brain in unprecedented ways but also actively manipulates neural circuits to probe their function in real time. Importantly, this interplay is beginning to inform the next generation of AI design. The limitations of current machine learning systems, such as catastrophic forgetting, inefficiency in sparse data settings, and hallucination, are not merely technical challenges but conceptual gaps that biological systems have evolved to solve. Through precise, AI-guided experimental neuroscience, we can uncover the regulatory, structural, and dynamical strategies the brain uses to achieve robustness, adaptability, and efficiency. Looking ahead, we propose a shift in mindset: from biologically inspired AI to biologically validated AI. Rather than taking metaphorical cues from the brain, future AI architectures may be grounded in mechanisms directly observed and tested in vivo. As AI continues to empower neuroscience, and neuroscience continues to inform AI, we are witnessing the emergence of a true feedback loop between mind and machine. This loop is not yet complete, but it is closing. With each cycle of innovation, we move closer to systems that not only compute but also reason, that do not just classify but understand. The promise of this convergence is not just smarter machines or better models, but a more profound, mechanistic grasp of intelligence itself, across both silicon and synapse. We look forward to seeing this mutually reinforcing relationship continue to evolve, fostering meaningful advances across both domains.

## Data Availability

No new data were created or analyzed in this study. Data sharing is not applicable to this article.
